# Strengthening Reinforced Concrete Beams through Integration of CFRP Bars, Mechanical Anchorage System, and Concrete Jacketing

**DOI:** 10.3390/ma17122794

**Published:** 2024-06-07

**Authors:** Mahmood Y. Alkhateeb, Farzad Hejazi

**Affiliations:** 1 Department of Civil Engineering, University Putra Malaysia, Serdang 43400, Malaysia; alkhateeb.m.y@gmail.com; 2 Department of Civil Engineering, School of Engineering, The University of the West of England, Bristol BS16 1QY, UK

**Keywords:** CFRP bars, strengthening, anchorage bolts, finite element analysis, flexural strengthening

## Abstract

The demand for strengthening reinforced concrete (RC) structures has increased considerably. Implementing carbon-fiber-reinforced polymer (CFRP) bars and concrete jacketing are the most effective techniques for RC beam retrofitting. Using the mechanical anchorage system (MAS) to attach CFRP bars to old concrete is highly recommended to avoid any debonding when it is applied to cyclic loads. However, the design of strengthening details is the most challenging issue because it involves many effective parameters. In this study, a design process for strengthening beams using CFRP bars with new MASs and concrete jacketing is proposed, and various design schemes are studied. The number of applied MASs and the thickness and grade of the concrete jacket were investigated through experimental testing and finite element (FE) simulations to define strengthening design details, such as the number and size of employed CFRP bars. Accordingly, an analytical technique was formulated to predict the performance of the strengthened beam in terms of the nominal ultimate load. The results demonstrated the high performance of the proposed system in preventing premature debonding. The proposed system enhances the beam capacity from 44 kN to 83 kN, representing an increase of more than 90%. In contrast, the conventional near-surface mounted (NSM) system exhibits a lower percentage increase at less than 37%. Both FE simulations and analytical approaches can be effectively employed to predict the behavior and capacity of the strengthened beam while considering various design parameters.

## 1. Introduction

Fiber-reinforced polymer (FRP) composite material is widely used in the strengthening process due to its desirable mechanical properties, such as high stiffness and strength-to-weight ratio, good creep and fatigue tolerance, resistance to corrosion and extreme environments, high strength, and elastic behavior until failure [1,2]. These properties encourage the use of FRP as an initial material for strengthening, especially in high-rise buildings, narrow structures where steel plates cannot be used, or where the structure cannot resist any additional dead load. Many techniques proposed using FRP depend mainly on attaching the FRP plate, sheet, or bars to the outside surface using an adhesive agent for bonding [3,4,5].

Conventionally, externally bonded (EB) and near-surface mounted (NSM) techniques are considered the optimum methods of strengthening RC beams [4]. The NSM method has several advantages over conventional EB; NSM rods are not exposed to the effects of the surrounding environment, such as fire and vandalism, and can be used on deformable surfaces, and the whole surface does not need to be prepared.

In NSM, the chance of premature debonding is low as the CFRP bars are totally embedded inside the grooves, and the outside surface of the beam remains aesthetically unchanged [6]. Furthermore, CFRP material shows comparative properties among other types of FRP materials because its tensile strength and elastic modulus are higher than those of GFRP and AFRP, leading to the use of smaller rod cross-sections and groove dimensions. The NSM system using CFRP Rode performs better than but still has several drawbacks.

The utilization of NSM bars significantly enhances the beam’s flexural strength, but it comes with inherent challenges. Shear strength may need to be increased during flexural strengthening to maintain critical flexural capacity. Flexural failures typically exhibit greater ductility compared with shear failures. In addition, the NSM technique poses constraints related to the need for sufficient solid concrete cover and the requirement of minimum edge and spacing clearances. Furthermore, concerns arise due to the relatively low resistance of CFRP to fire, the degradation of its properties under UV radiation, and potential incompatibility between the epoxy resin and the concrete substrate [7,8].

Abdallah et al. [9] proposed and examined the application of NSM methods using CFRP bars positioned on the side of the beam and affixed within pre-cut grooves situated laterally adjacent to the longitudinal steel reinforcement. Their experimental findings suggest that the mode of failure is influenced by the length of the CFRP bars and the properties of the filling material. Haryanto et al. [10] explored the impact of NSM design parameters on the performance of strengthened beams under monotonic and low-reversed cyclic loads. Their findings demonstrate the effectiveness of the strengthening system in capturing cracking and dissipating energy more efficiently. Moreover, the embedment depths of the CFRP bars significantly enhance the displacement ductility factor.

The usage of CFRP ropes is a relatively recent and cost-effective approach for strengthening and rehabilitating RC elements. Murad and Abu-AlHaj [11] explored the application of CFRP ropes to strengthen heat-damaged and undamaged beams through the NSM technique. The beams, reinforced with two continuous layers of CFRP bars, exhibited enhanced beam capacity and stiffness, as indicated by the test results. In addition, the utilization of CFRP material has been extended to retrofitting RC joints damaged under seismic loading. Existing studies have proposed various methods, such as the use of diagonally placed external CFRP ropes [12,13,14] or CFRP sheets [15,16] to strengthen RC joints. Golias et al. [17] conducted experimental investigations on a novel and efficient strengthening system employing X-type CFRP ropes and bonding the RC joint connection using CFRP sheets.

A mechanical anchorage system (MAS) is widely utilized to enhance the performance of strengthened beams with CFRP bars. Various novel anchorage systems have been proposed and investigated to mitigate premature debonding and improve system integrity. Recently, an innovative technique involving externally bonded materials in grooves was suggested to protect the strengthening material from surrounding environmental effects. In addition, a novel anchorage technique utilizing spike anchors on NSM bars was explored. This system had a more pronounced effect on the strengthened beam performance [18]. Also, three anchorage systems were developed using sisal-fiber-reinforced polymer (SFRP) to prevent the early delamination of the RC beams, namely, the SFRP U-wrap end anchorage (SE) system, SFRP U-wrap end and middle anchorage (SEM) system, and epoxy chemical bolt end anchorage (EE) system [19]. The SEM anchorage system was the most effective at preventing delamination, and the strengthening configuration (U-shaped) increased the ultimate load-carrying capacity of RC beams by a significant amount because it improved bonding at the side faces of the beams.

Guang et al. [20] suggested wrapping CFRP fabric on the outside surface of the CFRP bars to increase the interaction and bonding between the rods and the surrounding adhesive material. The attachment anchorage enhanced the strain in the CFRP bars and thus increased the beam capacity by 17.3% compared with the control beam. Zheng et al. [21,22] experimentally proved the effect of the proposed anchorage system, which used mainly CFRP anchorage, on enhancing the value of yielding and ultimate load. Furthermore, several attempts and proposed systems were developed to anchor the CFRP material [23,24]. Obaidat et al. [25] presented a new mechanical anchorage system that uses steel plates to fix the CFRP material at its end. The steel plates were attached to the top and bottom of the strengthened beam with anchorage bolts that extended between them. The test results proved that the existing anchorage systems are more efficient in improving the beam’s performance in terms of deflection, ductility, stiffness, and capacity. Esmaeili et al. [26] proposed an innovative plate anchorage technique, which uses two steel plates (fixed by welding through the angles) attached at the end of the strengthening material near the support. The plate anchorage system shows a comparative advantage in delaying or deleting the effect of the debonding problem compared with a number of suggested techniques in the literature. However, this system still has drawbacks, including FRP sheet rupture at the steel anchorage system’s edge due to local damage, lack of protection for FRP material from environmental effects, challenging installation procedures affecting field application efficiency, and unrealistic assumptions about the beam’s bottom surface condition in older structures. In addition, the system minimally affects the beam’s bottom and side views, which may not be preferable for structural aesthetics. Thus, improving local strengthening by using FRP sheets at the junction between FRP layers and steel plates could delay sheet rupture. Furthermore, adding a layer of concrete to protect the FRP and provide a smoother surface is recommended for enhanced durability and aesthetics.

Jacketing was utilized to improve the bearing capacity of structural members, reduce deflection, and restore the structural integrity of strengthened beams. This method is one of the most effective approaches for repairing and reinforcing beams by enlarging the beam section with an additional concrete layer and additional reinforcement [27]. Concrete jacketing not only increases the stiffness of the beam and its loading capacity but also reduces the stress at the crack tip to a value even lower than on the uncracked beam [28]. Ravel and Dave [29] investigated the effectiveness of eight methods of jacketing on smooth and chipped beam surfaces. The best bonding performance on a smooth surface requires added bonding agents and dowel connectors with micro-concrete; the same performance can be achieved with a chipped surface without using any additional connectors or even bonding agents. Hong and Lim [30] confirmed through experiments that the thickness of the concrete jacketing and the type of strengthening material have a significant effect on the mode of failure, stiffness, and resistance capacity of the strengthened beam. Ultra-high-performance fiber-RC is also recommended for use as concrete jacketing under quasi-static loading, enhancing the stiffness and strength compared with the concrete beam [31]. Thus, several studies have suggested and investigated different materials and grades for concrete jacketing, such as high-ductility cementitious composite [32], high-performance concrete, FRP composites [33], self-compacting concrete-filled jackets [34], fiber-reinforced self-consolidating concrete jackets [35], ultra-high-performance concrete strengthened RC beams [36], and high-strength engineered cementitious composite jacket [37].

In summary, various novel configurations have been proposed to mitigate the drawbacks of the NSM method, with a primary focus on addressing premature debonding issues. These innovations commonly involve introducing additional fixings to conventional systems, such as anchorage bolts [38,39,40] or end fixings [25]; suggesting modified configurations, such as side NSM [41,42,43,44] and CFRP rod panel [4,45,46,47]; or employing a cementitious material cover for the strengthening system [48,49]. Despite these efforts, both NSM and alternative systems still encounter challenges related to debonding between existing structures and the entire strengthening system or its individual components [50,51]. Early debonding remains a significant risk to the overall efficiency of these systems, particularly under conditions of vibration and repetitive loads.

The proposed system is developed to address challenges and drawbacks in previous systems, particularly premature debonding, effectively and durably. This approach integrates various techniques, including CFRP bars, MAS, concrete jacketing, and adhesive epoxy. The system primarily involves incorporating additional CFRP bars attached to the beam’s bottom surface. A MAS, comprising two steel plates connected to the beam with anchorage bolts, secures the CFRP bars. Subsequently, the CFRP bars and MAS are enveloped in concrete jacketing firmly bonded to the existing beam using epoxy adhesive material. This comprehensive system aims to enhance the overall effectiveness of the strengthening approach. Al-Mashgari et al. [52] applied the proposed system to four beams, which were subsequently constructed and tested experimentally until failure under increasing static loads. The testing protocol followed ASTM D6272 standards [53] for the four-point bending test of the RC beam. The ultimate load capacity and ductility increased by 32% and 52%, respectively. Nevertheless, the strengthened beam also experienced premature debonding between the beam surface and concrete jacketing, indicating the limited effectiveness of the bonding agent that was used. Therefore, adopting an alternative bonding agent is necessary to delay or eliminate premature debonding, thereby enhancing beam capacity more effectively. Furthermore, Alkhateeb et al. [54] investigated the performance of the strengthened beams under incremental repetitive bending moments. They found that the performance of the beams was enhanced by the new system compared with the control beam (CB) or even the NSM conventional system. The load-carrying capacity increased from 44 KN for the CB to 83 KN for the strengthened beam.

The main objective of this study is to introduce a proposed system for retrofitting RC beams, aiming to overcome the limitations of conventional strengthening methods. This system integrates CFRP bars, MASs, concrete jacketing, and bonding agents. The effectiveness of the system is experimentally evaluated under half-cyclic vibration load conditions. In addition, this study investigates the influence of various design parameters, such as CFRP rod diameter, the number of MAS units, and the grade and thickness of concrete jacketing, on the performance of the strengthened beams through the development of finite element (FE) models. Furthermore, analytical modeling is utilized to predict the ultimate load-bearing capacity of the strengthened beams.

## 2. Proposed Strengthening Scheme for RC Beams

**Figure 1:**
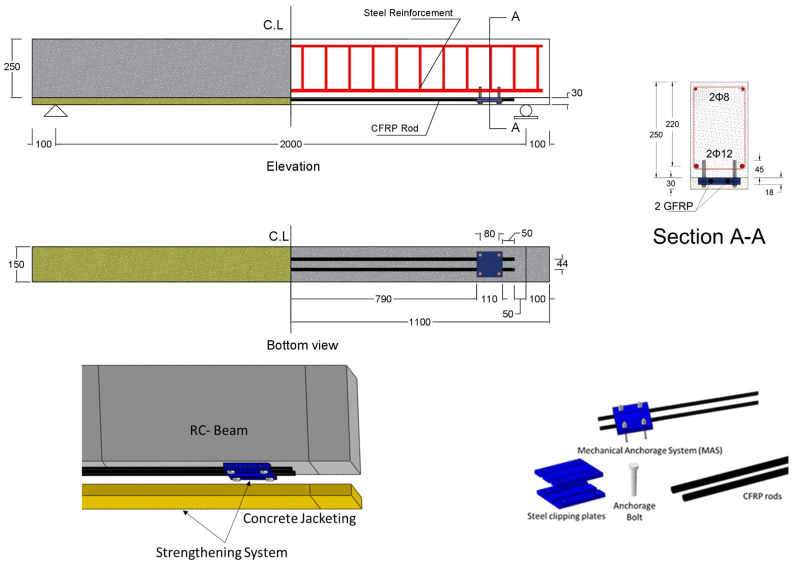
Configuration and main components of the proposed system. (All dimensions in mm).

The proposed system relies primarily on CFRP bars to enhance the beam’s flexural capacity, MAS to anchor the CFRP bars in place, concrete jacketing to protect the FRP material and anchorage system from surrounding effects, and bonding agent to prevent premature debonding between the existing beam’s concrete surface and the fresh concrete jacketing, as illustrated in Figure 1.

**Figure 2:**
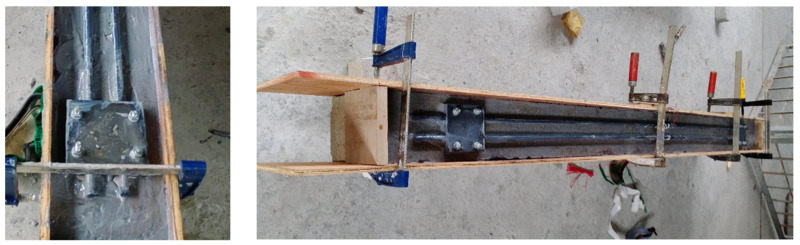
Strengthened beams before casting the concrete jacketing.

The CFRP bars are used to enhance the beam’s flexural capacity. The CFRP bars had a length of around 1900 mm and a diameter of 12 mm. The length of the CFRP rods was 50 mm from each support, adhering to the accepted anchorage length in line with design guidelines and recommendations such as ACI 440.2R-17 Chapter 14 [5]. Extending CFRP bars along the entire length of the beam, particularly under supports, which are typically within concrete columns, is a complex task. The MAS was used to fix the CFRP bars to the tension surface at the bottom of the beam, as shown in Figure 2.

**Figure 3:**
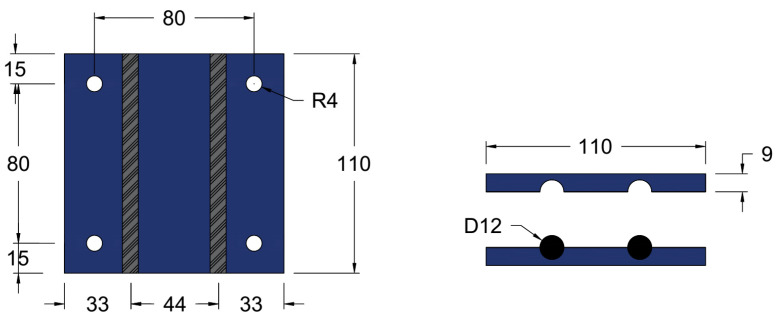
Dimensions of the proposed steel plate. (All dimensions in mm).

The MASs comprise steel clipping plates fixed to the bottom surface of the beam using four anchorage bolts, then CFRP bars are slotted onto the steel plates, and finally, another set of steel plates covers the bars. Each steel plate is manufactured as a square plate with dimensions of 110 × 110 mm, as shown in Figure 3. There are four holes in each plate; the center of these holes is located 15 mm from the steel plate edge. The radius of these holes was 4 mm, which is appropriate for the selected anchorage bolts. Two longitudinal channels were created on the plate surface to accommodate the CFRP bars. These channels had internally textured surfaces to introduce moderate roughness.

Considering the smoothness of CFRP bars, a friction coefficient of 0.3 to 0.4 was chosen to increase friction and prevent smooth sliding. The manufacturing tolerances for the grooves ranged from ±1.5 to ±3.0 mm. The circular shape of the CFRP bars was designed to match the grooves, ensuring a snug fit and secure anchorage within the mechanical system. Local failure of the CFRP bars was prevented through meticulous design and installation practices, ensuring adequate anchorage length and proper surface preparation for strong bonding to the surrounding concrete. This approach effectively distributed loads and reduced the risk of premature debonding.

The anchorage bolts fix the bottom steel plates, tidily close the top and bottom steel plates together after the CFRP bars are installed between them, and resist any stress developed from the CFRP bars’ movement. Four bolts are used for each pair of steel plates on one MAS. These bolts hang and fix these steel plates with CFRP bars to the beam’s bottom surface before casting the concert jacketing. In the experimental work, anchorage bolts with a total length of 70 mm were used: 40 mm was the bolt length inserted and expanded inside the concrete body, which was enough for fixation, and 30 mm was the part of the bolts outside the concrete. Of this, 18 mm served to secure the steel plates’ thickness (each with a thickness of 9 mm), while 7 mm accommodated the bolt’s washer height (with a thickness of 5 mm). The bolts’ diameter was 8 mm, which is optimum for the beam and the steel plate’s proposed design dimensions and provides the required resistance.

The required anchorage length of Hilti bolts, or any anchor bolts, is determined by factors such as bolt strength, base material (concrete) strength, applied loads, and stress type (tension, shear, or combined). Hilti offers specific guidelines and software tools for their products. In this study, HS 70/10/20 anchorage bolts were selected based on Hilti’s guidelines. Table 1 outlines the key characteristics of the bolts.

**Table 1:** Characteristics of the Hilti anchorage bolts.

Effective anchorage depth h_ef_	70 mm	Mechanical properties		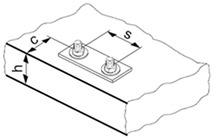
Design resistance		Nominal tensile strength f_uk,thread_	580 N/mm^2^	
Tension N_Rd_	10.7 kN	Yield strength f_yk,thread_	464 N/mm^2^	
Shear V_Rd_	8.5 kN	Stressed cross-section As	36.6 mm^2^	
Recommended loads		Moment of resistance W	31.2 mm^3^	
Tension N_rec_	7.6 kN	Char. bending resistance	21.7 Nm	
Shear V_rec_	6.1 kN			

Typically, the anchorage length for a bolt can be calculated using a simplified formula.

(1) $$l_{a}=\frac{N_{b}\times \emptyset }{f_{bd}}$$

where

$l_{a}$ is the anchorage length.

$N_{b}$ is the design load per bolt.

∅ is a coefficient considering the conditions of the bond, the presence of reinforcement, etc.

$f_{bd}$ is the design bond strength.

Adhering to specifications from the Hilti datasheet is crucial to ensure anchorage system integrity and safety. Key parameters include a minimum base material thickness (h_min_) of 120 mm, a minimum spacing between bolts (s_min_) of 35 mm, and a minimum edge distance (c_min_) of 35 mm from the center of bolts holes to the edge of the concrete member. In the current proposed system, the distance from the center of the holes to the edge of the steel plate is 15 mm, and the distance from the edge of the steel plate to the edge of the beam is 20 mm. Thus, the total distance from the center of the holes to the edge of the beam is 35 mm, which aligns with the specified conditions. Critical condition analysis aids in selecting appropriate dimensions for anchorage bolts and steel plates, considering factors such as applied loads and material properties, thus ensuring optimal performance and reliability under various loading conditions.

Precise control over bolt torque was ensured during installation to mitigate the risk of CFRP bar crushing under transverse loads. This process involved adhering strictly to specified torque values provided by the manufacturer (Hilti) and employing torque wrenches for accurate bolt tightening.

Concrete jacketing is used to provide the necessary medium to transfer the stress from the beam to the attachment CFRP bars and provide a proper cover for the CFRP materials, thereby reducing environmental and other effects. The proposed thickness of the concrete jacketing was 30 mm, which provided the required cover for the other strengthening components; however, the optimum thickness and grade of the concrete are investigated in this study. Additional epoxy adhesive material is used to connect the two concrete surfaces, which completely prevents the premature debonding of the proposed system and ensures its effective performance.

## 3. Beam Considered for Strengthening Using CFRP Bar, MAS, and Concrete Jacketing

In this study, a simply supported reinforced concrete beam was selected for strengthening. The experimental work was conducted under incremental repetitive (half-cycle as pushing and resting) loads as premature debonding is more likely to occur under repetitive loads; thus, the validation FE models were simulated under the same loads. However, the FE parametric study models and the analytical equations were created under incremental static load because the current standard code’s formula was developed under static load.

### 3.1. Geometric Details

**Figure 4:**
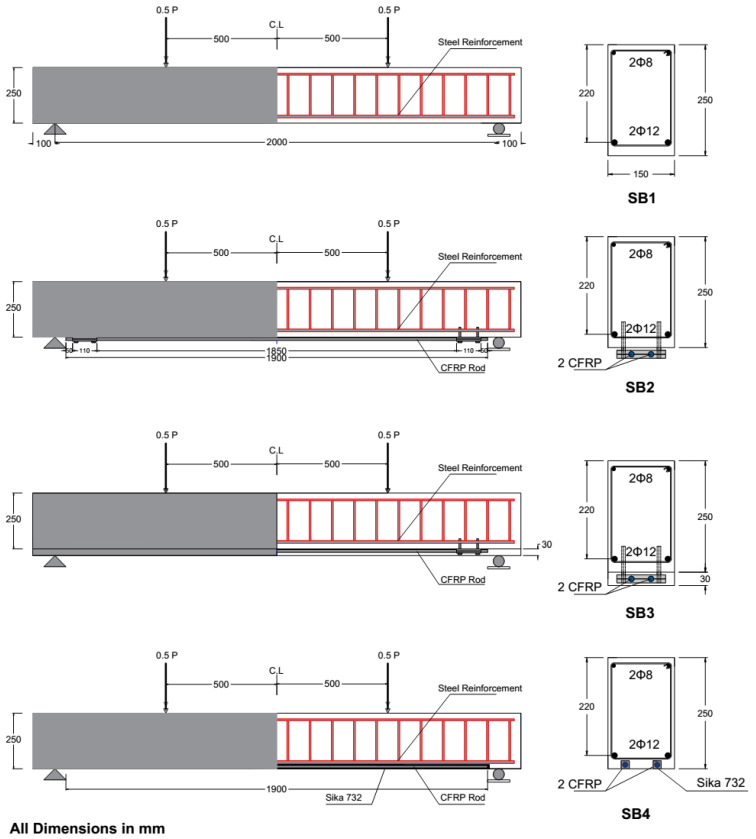
Beam geometry details. (All dimensions in mm).

A reinforced concrete beam with a 2000 mm clear span length and a 150 × 250 mm cross-section was adopted. Two 12 mm steel bars were implemented as the main reinforcement, located at an effective length of 220 mm. At the top, two bars with an 8 mm diameter were installed. Closed stirrup bars with an 8 mm diameter and 100 mm space were used to provide the required shear resistance for the existing beam before attaching the strengthening system. However, they may not suffice after strengthening the beam and increasing its flexural capacity. A 20 mm concrete cover was considered for the beam on all sides except for the bottom, which was 24 mm thick. A total of four beams were constructed and tested experimentally under incremental repetitive (half-cycle) loads. Table 2 shows the constructed beam labeling, and Figure 4 demonstrates the main specifications of these four beams.

**Table 2:** Details of constructed beams.

Beam	Beam Labeling	Method of Strengthening
Control beam (reference beam)	SB1	-
Strengthened beam with 2 CFRP bars, without concrete jacketing	SB2	2CFRP + 2MAS + NCJ
Strengthened beam with 2 CFRP bars and concrete jacketing	SB3	2CFRP + 2MAS + CJ
Strengthened beam with 2CFRP according to NSM techniques	SB4	2CFRP + NSM

CFRP: CRRP bar; MAS: mechanical anchorage system; NCJ: not including concrete jacketing; CJ: concrete jacketing; NSM: near-surface mounted.

The number of specimens for testing was limited due to budget constraints from the allocated funds. For this reason, the experimental work was performed after an extensive simulation study using the FE program to validate the results. The significant agreement between the experimental results and the FE simulation output validated the experimental test output. However, a greater number of testing specimens could lead to more reliable results.

### 3.2. Material Properties

**Figure 5:**
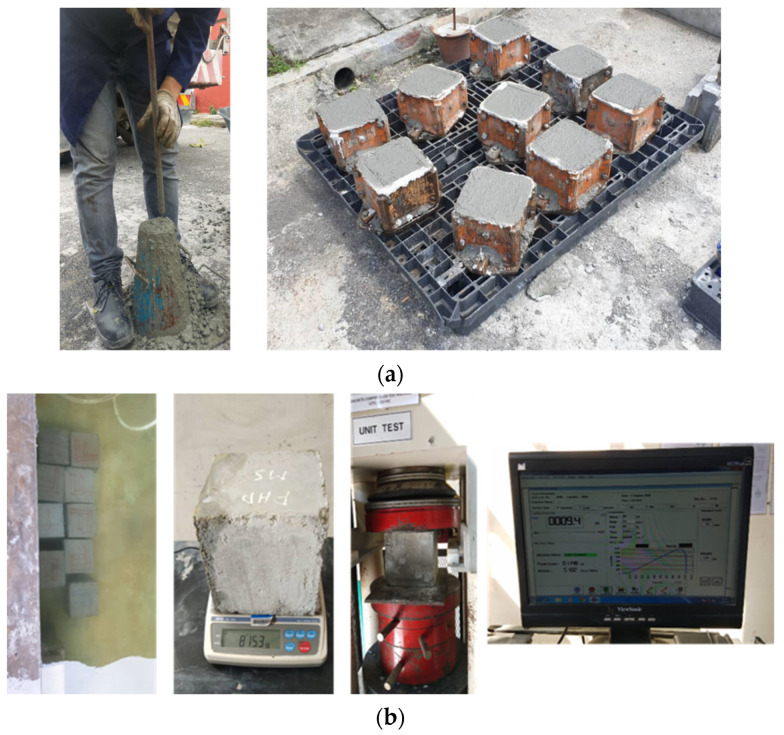
Concrete mix property evaluation tests: (**a**) slump test for fresh concrete and cube samples; (**b**) curing and testing of concrete cubes.

All the beams in the experimental program were made from a single patch of ready-mix concrete. The compressive strength of the concrete was set at 40 MPa, and the required mechanical property tests for concrete were conducted to ensure its properties, as shown in Figure 5. The concrete compression test results after 28 days are listed in Table 3. The jacketing material strength utilized in the test was 40 MPa. The concrete jacketing was cast in the materials laboratory, and the required tests for the mechanical properties of concrete were conducted, with their results listed in Table 3. The yield strength and modulus of elasticity of the main and transverse reinforcements were 550 MPa and 210,000 GPa, respectively. The mechanical properties of the utilized steel bar were obtained from the factory and supplier’s specifications sheet. Additionally, a few steel samples were tested in the lab using a universal tensile machine (UTM) to verify adherence to the standard and corroborate the information provided by the factory, as shown in Table 4. 

**Table 3:** Results of concrete compressive strength after 28 days.

	Beam Concrete	Concrete Jacketing
Cubic 1	45.9	45.9
Cubic 1	48.17	46.15
Cubic 1	45.07	45.1
Cubic 1	44.44	42.12
Cubic 1	47.2	49.13
Cubic 1	50.78	44.32
Average after 28 days	46.2	45.3

**Table 4:** Steel bar tensile test.

	Yield Strength MPa	Ultimate Tensile Strength MPa
Steel bar 1	558	671
Steel bar 2	554	665
Steel bar 3	564	675
Average value	558.67	670.33

**Figure 6:**
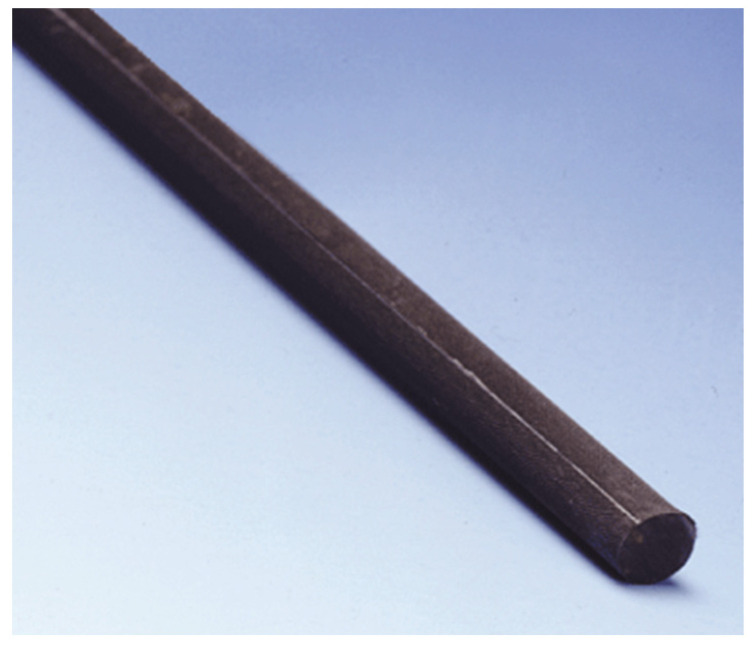
Maperod C.

The CFRP bars were obtained from MAPE Production (Maperod C) [55], as shown in Figure 6, with an ultimate tensile reach of 2000 MPa, a modulus of elasticity of 155,000 GPa, and a maximum elongation at failure of 0.015.

The mechanical anchorage system consists of steel plates produced in a local workshop using S275 steel material and Hilti anchorage bolts sold under the product name HAS expansion anchorage bolts M8/70/20/10 [56]. Sikadur^®^-732, an epoxy resin bonding agent [57], was used to create the required bond between the old and fresh concrete surfaces. The required mechanical properties of the used epoxy based on manufacturing datasheets are shown in Table 5.

**Table 5:** Mechanical properties of epoxy adhesives.

Compressive strength (MPa)	63 (ASTM D-695) [58]
Tensile strength in flexure (MPa)	39 (ASTM D790) [59]
Tensile adhesion strength (MPa	2
Mixing ratio	Comp. A:B = 2:1 by weight/volume
Consumption	0.3–0.8 kg/m^2^, depending on substrate condition
Pot life	35 min (at +30 °C)

### 3.3. Casting Concrete Beams

**Figure 7:**
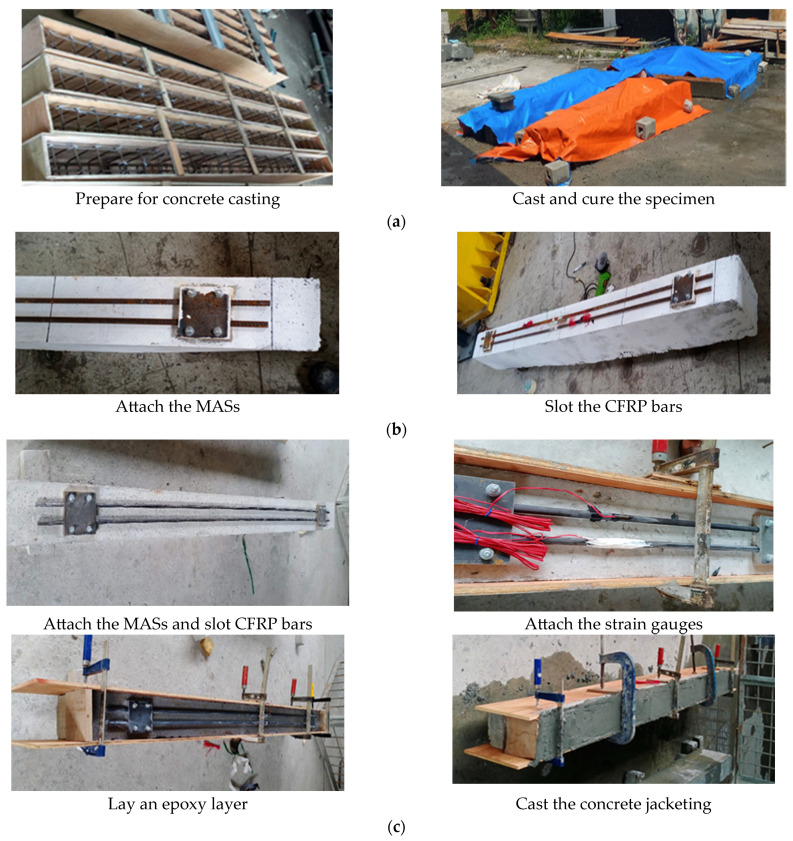
Casting concrete beams and attaching the strengthening system: (**a**) casting the concrete beams; (**b**) setting the strengthening system for SB2; (**c**) setting the strengthening system for SB3.

Figure 7 illustrates the main procedure for casting the concrete beams and attaching the configured strengthening systems. The beams were prepared for casting after locating the steel reinforcement and strain gauges inside the wooden modules. KYOWA strain gauges were utilized to measure the strain for the flexural rebar reinforcement and the additional strengthening CFRP rods. The KFGS-10-120-C1-11 model was used with a total length of 10 mm and gauge resistance of 119.8 Ω ± 0.2% at a temperature of 23 °C and humidity of 50% RH. The strain gauges were attached in the middle of each steel bar. After concrete curing, the strengthening system was installed by fixing the bottom steel plates in their location on the beam bottom surface, slotting the CFRP bars on the steel plates and closing them by the top steel plates, placing the strain gauges in the middle of CFRP bars, applying epoxy on the beam surface, and casting the fresh concrete jacketing. The laboratory’s adhesive application procedure adhered to stringent guidelines and proceeded as follows: First, the substrate concrete surface underwent meticulous preparation to guarantee cleanliness and structural integrity, with thorough removal of any contaminants such as dust, laitance, grease, curing compounds, impregnation, waxes, foreign particles, or disintegrated materials. Sandblasting was utilized to achieve the desired surface texture on the concrete. Subsequently, Sikadur^®^-732 was meticulously prepared by thoroughly mixing two parts Component A with one part Component B in a clean pail for 3 min using a low-speed drill until a uniform color was achieved. The blended Sikadur^®^-732 was then evenly applied onto the substrate using a brush, ensuring consistent coverage. Fresh concrete was promptly applied onto the adhesive while it remained tacky, facilitating a robust bond with the hardened concrete.

### 3.4. Loading and Experimental Testing

The load was applied using an MTS hydraulic jack equipped with a servo valve capable of handling loads up to 1000 kN. Positioned vertically within the supporting frame, the jack’s actuator was controlled using a 4830 digital smart Shimadzu Servo Controller. The cyclic load is applied by pushing and pulling. However, for the beam, this process involved an unloading phase after each pushing step, allowing the jack head to return to the zero position (rest phase). Subsequently, the cycle was repeated, progressively displacing the beam further and then returning the jack to the zero position. This method is referred to as a half-cycle, comprising incremental cyclic pushing and pulling back to the zero condition.

**Figure 8:**
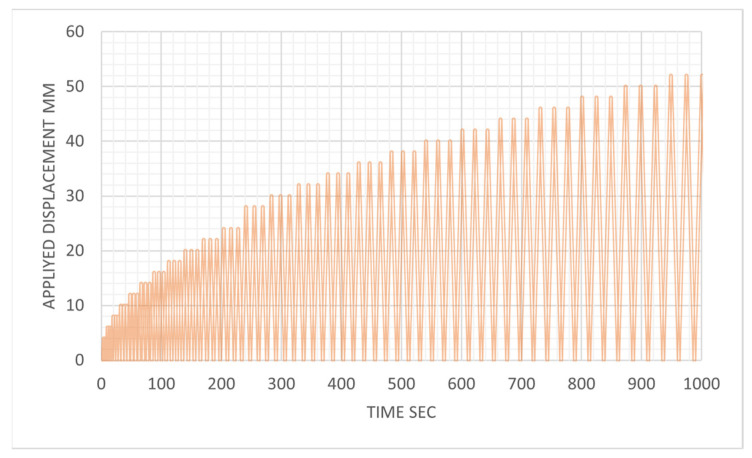
Loading protocol based on ACI committee 374.1-05 ATC (1996).

The experimental tests were conducted using a displacement control method, in which a displacement time history was predefined and inputted into the controller. The controller then maneuvered the actuator in accordance with this timeline, simultaneously recording the necessary load to achieve the displacement at each 0.01 s interval. The loading protocol applied to the beam was formulated based on ACI 374.1-05 ATC (1996). Each displacement magnitude was iteratively replicated three times, as illustrated in Figure 8, to ensure beam stable behavior with a loading rate of 2 mm per second.

**Figure 9:**
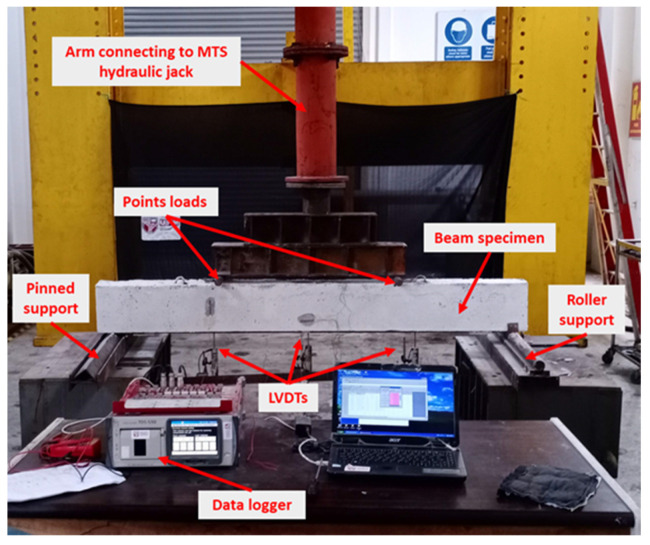
Experimental sitting for testing the strengthened beam specimens.

The setting of the specimen is shown in Figure 9. The specimens are positioned over rollers and pinned supports, enabling them to behave as simply supported beams. The load was applied through a vertical hydraulic arm, which was applied to the beams through two points at the top of the beam. These two points are located 500 mm from each support. Load and displacement data for the dynamic actuator were precisely measured using a load cell and a linear variable differential transformer (LVDT) located within the jack’s head, with data capture facilitated by the servo controller. The servo controller primarily generates specified displacements according to the protocol load and records the corresponding load required for generating them. Extra LVDTs were strategically installed at various points on the beam to measure and monitor its deformation throughout the testing process. The LVDTs and strain gauges were connected to the data logger device, which captures and records the data obtained from them.

## 4. Development of Finite Element Model

An FE model was developed to investigate the RC beams’ behavior strengthened by the proposed system and the parametric studies. The general-purpose FE software Abaqus 6.14 [60] was used to perform the nonlinear analysis. Two groups of FE models were generated. The first group of beam models was used to validate the experimental specimens, while the second group of models was dedicated to investigating the design parameters.

The FE simulation mainly involves part generation, material definition, assembly, interaction, mashing, loading, boundary conditions, and analysis.

### 4.1. Material Properties for FEM Simulation

In the FE simulations, several types of materials were defined and applied on the different model-generated parts. The properties of these materials are demonstrated here.

#### 4.1.1. Concrete

In Abaqus, a 3D deformable solid element can be used to simulate concrete beam specimens. For all beams, a concrete grade of 40 MPa was utilized, while varying grades of 25, 40, and 80 MPa were applied for the concrete jacketing to assess the impact of its compressive strength on overall performance. The mechanical properties for the different concrete grades, including compression strength ($f_{c}^{\prime }$) modulus of elasticity ($E_{c}$), Poisson’s ratio (v), and tensile strength ($f_{t}$), are listed in Table 6.

**Table 6:** Concrete material properties.

Concrete Type ID	Concrete Compressive	Elastic Modulus	Poisson’s Ratio	Tensile Strength
	$\mathrm{Strength}\;f_{c}^{\prime }$ (MPa)	$E_{c}$ (GPa)		$f_{t}$ (MPa)
Type (1)	25	23.50	0.2	3.1
Type (2)	40	29.73	0.2	3.9
Type (3)	80	42.04	0.2	5.5

The concrete modulus of elasticity and tensile strength are calculated according to ACI 318-19 standard coding [61] using Equations (2) and (3).

(2) $$E_{c}=4700\sqrt{f_{c}^{\prime }}$$

(3) $$f_{t}=0.62\sqrt{f_{c}^{\prime }}$$

Concrete properties are assumed to be isotropic during the elastic phase, and the concrete damage plasticity (CDP) model was employed to simulate the plastic phase’s material behavior [62,63]. This model incorporates a combination of isotropic damaged elasticity and isotropic tensile and compressive plasticity, representing the concrete’s inelastic behavior, whether plain or reinforced. The primary failure mechanisms under load include tensile cracking in tension and concrete crushing in compression [64].

Utilizing the CDP model to represent the nonlinear behavior of concrete in FE modeling involves specifying parameters for concrete plasticity, compression behavior, and tension behavior. Material parameters known as plasticity flow parameters must be identified to define plasticity. These parameters include dilatation angle (Ψ), flow potential eccentricity (∈), stress ratio ($\sigma_{bo}/\sigma_{co}$), and ($K_{C}$). The dilatation angle (Ψ) characterizes internal friction within concrete. In 3D solid finite element analysis, values for (Ψ) are chosen carefully, guided by a comprehensive review, empirical data, and calibration. Recommended within the range of 20 to 45 [65,66], (Ψ) was set to 35, aligning with best practices for accurate simulation of concrete behavior. The flow potential eccentricity (∈) is a small positive number that determines how rapidly the hyperbolic flow potential approaches its asymptote. In this work, a value of 0.1 was chosen [60]. The default value of 1.16 was used for the biaxial compressive yield stress to the uniaxial compressive yield stress (stress ratio $[\sigma_{bo}/\sigma_{co}$]). The parameter ($K_{C}$) was set to its default value of 0.6667, with values ranging from 0.5 to 1.

**Figure 10:**
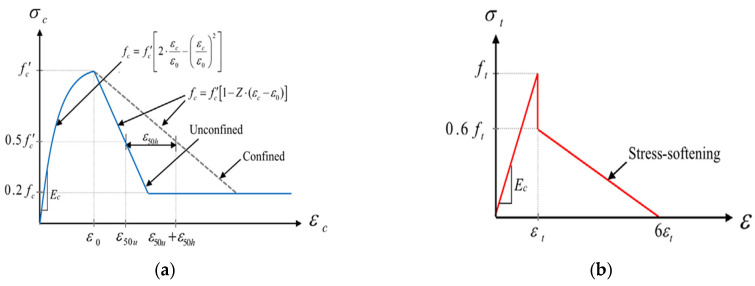
Stress–strain models: (**a**) concrete in compression; (**b**) concrete in tension.

The stress–strain curve relationship, either compressive or post-cracking tension, developed by Hognestad [67] for the ascending part and the descending (softening portion) developed by Kent and Park [68] were selected for this study. Figure 10a shows the data that should be entered into the software for concrete in compression, calculated based on Equations (4) and (5). Equation (4) is applied in the first region when the strain in the concrete is less than the strain at the maximum stress ($\varepsilon_{o}$), while Equation (5) is used to present the linearly descending curve, either for confined or unconfined concrete in the second region where the strain in the concrete is more than ($\varepsilon_{o}$) but less than the maximum strain ($\varepsilon_{cu}$). Equation (5) in the second region is based on deterioration constant (Z) that adjusts for the effect of the strain on concrete strength and controls the slope of the line.

(4) $$f_{c}=f_{c}^{\prime }\left[2\cdot \frac{\varepsilon_{c}}{\varepsilon_{o}}-{\left(\frac{\varepsilon_{c}}{\varepsilon_{o}}\right)}^{2}\right]\;where\;\;0\leq \varepsilon_{c}\leq \varepsilon_{o}$$

(5) $$f_{c}=f_{c}^{\prime }\left[1-Z\cdot \left(\varepsilon_{c}-\varepsilon_{o}\right)\right]\;where\;\;\;\;\;\;\;\;\;\;\varepsilon_{c}>\varepsilon_{o}$$

Figure 10b shows the stress softening model for the concrete in tension. The stress softening model was adopted in this work because stress softening is particularly significant in cyclic loading or dynamic loading situations, where repeated loading and unloading cycles can exacerbate the development of microcracks and further degrade the concrete’s mechanical properties over time.

In Abaqus, defining concrete compression behavior involves inserting the strain–stress date of the inelastic stage. In simulating concrete tension behavior, the strain–stress value of the cracked stage also needs to be inserted. To obtain concrete compression and tension damage, a sub-option needs to be defined with the compression damage parameter (*dc*) and its corresponding inelastic strain, and tension damage parameters (*dt*) with the cracking strain. Damage variables (*dc*, *dt*) in Abaqus represent the progression of material damage in concrete under various stresses. Their values depend on the load rating, with a slower rate yielding more reliable results. Typically modeled using exponential or power-law functions, these variables quantify the deterioration in tensile and compressive strength. Several equations have been developed to calculate elastic and inelastic strain in compression ($\varepsilon_{oc}^{el},\varepsilon_{c}^{il}$), elastic and inelastic cracking in tension (${\varepsilon_{ot}^{el},\varepsilon }_{t}^{il}$), and compression and tension damage parameters (*dc*, *dt*). However, in this study, Equations (6)–(8) were applied to calculate the required values for input into the program.

(6) $$\varepsilon_{oc}^{el}=\frac{f_{c}}{E_{c}}\;\;\;\;\;\;\;\;\;\;\;\;\;\;\;\;\;\;\;\;\;\;\;\;\;\;\;\;\;\;\;\;\varepsilon_{ot}^{el}=\frac{f_{t}}{E_{t}}$$

(7) $$\varepsilon_{c}^{il}=\varepsilon_{c}-\varepsilon_{oc}^{el}\;\;\;\;\;\;\;\;\;\;\;\;\;\;\;\;\;\;\;\;\;\;\;\;\;\;\;\;\varepsilon_{t}^{il}=\varepsilon_{t}-\varepsilon_{ot}^{el}$$

(8) $$d_{c}=1-\frac{f_{c}}{f_{cu}}\;\;\;\;\;\;\;\;\;\;\;\;\;\;\;\;\;\;\;\;\;\;\;\;\;\;\;\;d_{t}=1-\frac{f_{t}}{f_{tu}}$$

#### 4.1.2. Steel Reinforcement

The longitudinal and stirrup steel reinforcements were modeled as 3D deformable wire parts. A classical elastic-plastic material with strain hardening based on von Mises plasticity was used to define the steel bar’s behavior. Thus, the bilinear stress–strain curve was adopted to define the reinforcing bar behavior in the program [69]. The modulus of elasticity in the elastic stage was 210 GPa, and the Poisson’s ratio was 0.3. The plastic behavior was defined by a yield stress of 550 MPa, with an ultimate stress of 625 MPa.

#### 4.1.3. CFRP Bars

The CFRP bars were developed with 3D deformable solid parts. CFRP is thought to be a purely elastic material because it behaves in a linearly elastic manner until brittle failure. In the fiber direction, the modulus of elasticity was 155 GPa, and the Poisson’s ratio was equal to 0.3, as specified by the manufacturer [55]. Thus, a linear up-to-failure stress–strain relation exists for the CFRP. Moreover, the failure strain was equal to 1.5%, as stated in the product datasheet.

#### 4.1.4. Adhesive Bonding Agent

In the experimental work, Sikadur^®^-732 [57] was selected to bond the old concrete surface with the new jacketed concrete surface. Thus, on the basis of the product sheet, the Poisson’s ratio can be taken as 0.37, the elastic modulus was 4.48 GPa, and a tensile strength of 24.8 MPa can be used to define the epoxy material’s properties.

#### 4.1.5. Mechanical Anchorage System

Simulating the mechanical anchorage systems in this study is challenging due to the relatively small size of the MAS compared with the beams, requiring careful consideration of various details. Moreover, this system involves smaller components that interact with each other to provide the required performance. Such interactions, besides the material and geometry nonlinearities, impose further nonlinearity during the analysis and slow convergences. Steel plates and anchorage bolts are the two main components in the MAS, and their simulation will be described below.

- Steel plates

A different steel material type was defined for the steel plates that are used in the MAS. For the steel plate, an elastic, perfectly plastic steel model was defined with a 550 MPa yield value.

- Expansion anchorage bolts

The bolts’ main bodies were simulated as 3D deformable evolution parts and needed to be pre-tensioned before applying the load. However, if the bolts’ main function in this work is to fix the two halves of the steel plates tidily together and to the concrete surface, then the anchored bolt modeling can be ignored and replaced by creating a tied interaction between the steel plate surfaces and the external surface of the concrete to reduce the simulation time.

#### 4.1.6. Loading and Supporting Parts

For the other steel components that are used as supports and to apply the load to the beam, a different steel material was defined as a rigid steel material. These components were designed to behave elastically until the end of applying the load under any load conditions. The properties of this rigid material were assigned as a fully elastic steel material with a 210,000 MPa Young’s modulus and a 0.33 Poisson’s ratio.

### 4.2. Assembly

Various parts were arranged in specific configurations to generate different beam models. Thirteen models were developed with different setups. In the assembly of the SB3 model, for example, the beam was composed of several parts: the RC beam, two top bars, and two bottom bars. Each bar was precisely positioned within the RC beam component. In addition, 21 closed stirrups were placed around them, spaced 100 mm apart.

The strengthening system in SB3 requires a certain arrangement. The two steel plates were pressed together, surface to surface. Four anchorage bolts were used to close these two plates and fix them to the beam’s bottom surface at their intended location. Between these two steel plates, the two CFRP bars were slotted. All these strengthening components—steel plates, anchorage bolts, and CFRP bars—were in their designated locations inside the concrete jacketing element. The concrete jacketing was connected to the beam’s bottom surface by anchorage bolts and a layer of epoxy.

### 4.3. Interaction

Various types of bonding and surface interactions were defined to ensure that the behavior and interaction of these parts in the models adequately reflect the real behavior observed in the experimental work. Perfect bonding between the concrete and reinforcing bars was simulated using the embedded region constraint model, which is a feature available in the program. Consequently, the steel bars were modeled as embedded regions within the concrete host, ensuring a realistic representation of their interaction. Various bonding scenarios can be explored to model the interaction between the old concrete surface and the new jacketing surface. However, a perfect bond was applied using a tie constraint contact, thereby preventing debonding from occurring between these surfaces, to adequately simulate the experimental conditions. The epoxy layer was further defined as a cohesive element, incorporating the traction–separation law to adequately capture its behavior.

Several assumptions were made to simplify the modeling process. Given that the anchorage bolts secure the two steel plates tightly together, the contact surface between the plates can be simulated using a tie constraint interaction, or alternatively, the two plates can be treated as a single part (a box plate). Moreover, because the anchorage bolts fix the steel plates to the beam surface, the contact between the plates and the beam was modeled using a tie constraint while considering the influence of the anchorage bolts. These simulation strategies ensure a satisfactory global response of the beam, achieving a reasonable agreement compared with the experimental results.

Finally, several interactions regarding the CFRP bars require precise delineation. Initially, the bond between the CFRP rods and the internal surface of the steel plates in SB2 was modeled as an interaction, incorporating friction coefficients of approximately 0.3. In contrast, in SB3, this bond was characterized as fully tied, as both the steel plates and CFRP bars were fully embedded within the concrete jacketing, functioning as a unified entity. Second, the interaction between the CFRP bars and the surrounding concrete jacketing was simplified as an embedded constraint for analytical expediency. Lastly, a distinct interaction was formulated for SB2 when the applied loading reached a specific threshold, causing differential displacement between the bottom surface of the RC beam and the CFRP bars. In such cases, frictionless interaction properties were deemed appropriate, reflecting the real-world scenario where the beam presses downward on the CFRP bars without inducing stress within the CFRP rod material. Additionally, two critical interactions were defined in SB4. The CFRP–epoxy interaction was modeled as a cohesive interaction to accurately represent the bond between the CFRP rods and the surrounding epoxy layer, with the CFRP rods fully embedded within the epoxy material. The epoxy–concrete interaction was applied at the interface between the concrete on the internal surface of the grooves and the epoxy inside the groove. Based on experimental evidence of bond failure, this interaction was defined using cohesive elements.

### 4.4. Loading and Boundary Condition

**Figure 11:**
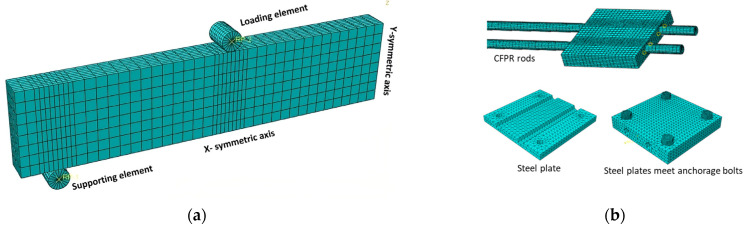
Strengthening beam meshing: (**a**) quarter symmetric model; (**b**) mesh for strengthening components.

Only one-quarter of the strengthened beams were developed during the FE modeling, taking advantage of the symmetry in both directions, where X-symmetric and Z-symmetric conditions were applied to the model, as shown in Figure 11a. Regarding the kind of support, a steel plate was attached at the bottom of the RC beam part or concrete jacketing part at 100 mm from the outside edge. This steel plate was defined as the roller support. Another loading steel plate was located at the top surface of the RC beam 500 mm from the supporting point. Two reference points were used to apply the loading and boundary conditions to prevent stress concentration at one point. The FE simulation has two load scenarios: an incremental half-cyclic analysis to validate the experimental test by mirroring the experimental test setup and load protocol in Figure 8 and an incremental static loading with a displacement control method for the parametric study.

### 4.5. Mesh

Different meshing techniques were used in these models according to the shape and size of the part. The holes for the anchorage bolts on the steel plates and the RC beam required special attention, and a series of partitions were used to regulate the mesh and avoid element degeneration. The initial meshing size was adopted based on several previous studies and the recommended values proposed by them, such as [60,63,70]. There are 4873 elements (C3D8R) for the RC beam part with a meshing size of 30 and the concrete jacketing with a meshing size of around 460 elements. There are 128 elements (T3D2) for the top-bar and bottom-bar steel reinforcement; however, there are 204 elements (T3D2) for stirrups. For each steel plate, 500 elements were created. A total of 588 elements (C3D8R) were proposed for the CFRP bars, with 300 elements for the anchorage bolts and 500 for the epoxy layers. Figure 11a depicts the meshing for one-quarter of the strengthened beams, while Figure 11b illustrates the meshing for the strengthening components.

Three mesh sizes were examined for the RC beam elements to assess the convergence of the numerical solutions. Comparisons were performed between the results obtained from each mesh size and the experimental data, focusing on the load–displacement curve. On the basis of these comparisons, a mesh size of 30 was chosen to achieve a balance between precision in output results and computational efficiency. However, the mesh size for the RC element in SB4 was intentionally selected to be larger than that of other beams. This decision was made because SB4 lacks concrete jacketing and other smaller parts for the MAS, which typically require more detailed meshing for accurate results.

## 5. Results and Verification

### 5.1. Load–Displacement Response

The ultimate load on beam SB1 (reference beam) was around 44 kN, which increased slightly to 48 kN on strengthened beam SB2 without concrete jacketing. In SB3, which represents the proposed system, the ultimate load was increased to 83 kN, and finally, the maximum load registered experimentally on SB4 was 60 kN. These values present the efficiency of using the proposed system to enhance the beam strength capacity even compared with conventional NSM (SB4).

**Figure 12:**
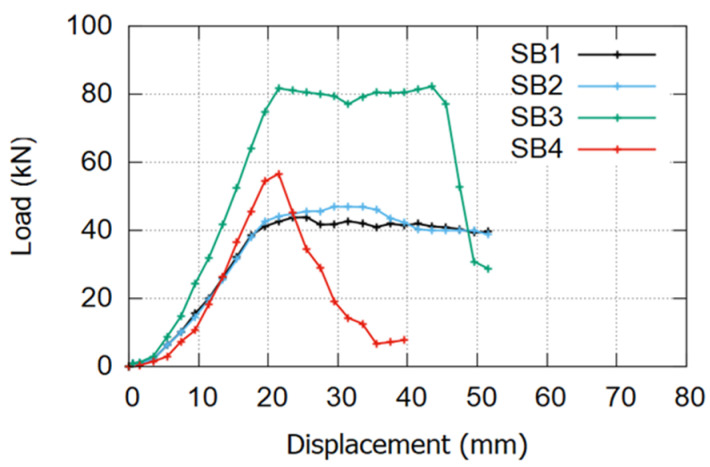
Load vs. displacement curve for experimental specimens.

Figure 12 shows a comparison between the experimental skeleton curve of the load versus displacement. The load–displacement curves presented in Figure 12 were obtained through a meticulous process, transforming the hypothetical relationship between applied load and displacement into a skeleton curve by selecting the peak point at the top of each increment. These loads include incremental pushing and resting phases. The strength of the beam under repetitive loads deteriorates due to the presence of existing cracks from previous cycles, which can propagate new cracks under cyclic loading. Consequently, the load-bearing capacity of the beam in each cycle is slightly reduced than when a load is applied in static conditions, which typically leads to linear behavior at the start of the test within the elastic range.

**Figure 13:**
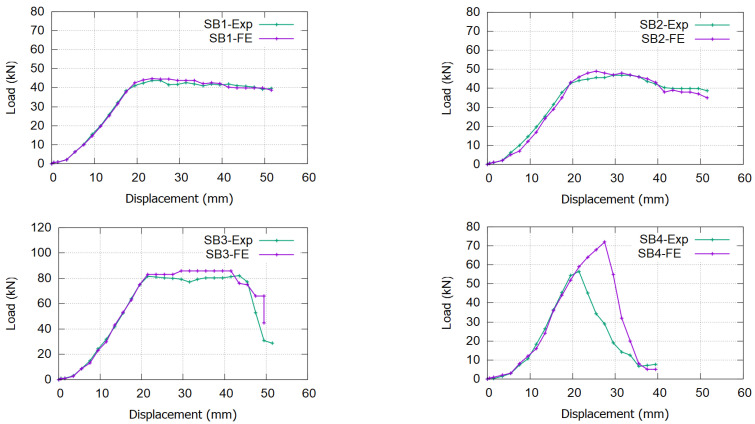
Load vs. displacement curve for the validation FE models compared with the experimental specimens.

The four FE models’ load versus displacement curves show good agreement with the experimental results, as shown in Figure 13, except for a considerable difference at SB4 (increasing the ultimate load from 60 kN experimentally to 74 kN in the modeling). The sudden debonding failure observed in SB4 during the experimental test was not adequately captured by the software due to inherent assumptions regarding material and interaction variables in the modeling process. Furthermore, the interaction surface in SB4 was defined twice—first, between the CFRP bars and the epoxy layer and, subsequently, between the epoxy and the concrete on the inner surfaces of the grooves. These complexities in defining interaction surfaces and limitations in modeling material behavior contributed to the discrepancy between the experimental and simulated results for SB4. The ultimate load on SB3 models was 10% higher than the experimental results. The FE corresponding displacement (33 mm) differed by almost 22% from the experimental one (27 mm), which can be attributed to the challenge of exactly replicating the material’s behavior in the software versus its actual performance in the experimental laboratory.

### 5.2. Ductility of the Beam Specimens

Ductility represents the plastic deformation of a beam measured from the yielding point without noticeable losses in strength. The values of displacement at yield ($\Delta_{y}$), at ultimate ($\Delta_{u}$), and at failure ($\Delta_{f}$) displacement are extracted for each beam from the load vs. displacement curve in Figure 12 and listed in Table 7. Yielding displacement ($\Delta_{y}$) is defined as the point where material deformation transitions from elastic to plastic. Ultimate displacement ($\Delta_{u}$) corresponds to the maximum load-bearing capacity, marked by the peak in the load–displacement curve. Failure displacement ($\Delta_{f}$) is the point where the specimen can no longer sustain a load, indicated by a significant drop in the load–displacement curve. Equations (9) and (10) were used to calculate two displacement ductility indexes ($\mu_{\Delta u}$) and ($\mu_{\Delta f}$). All the calculations of the ductility index are summarized and presented in Table 7.

(9) $$\mu_{\Delta u}=\frac{\Delta_{u}}{\Delta_{y}}$$

(10) $$\mu_{\Delta f}=\frac{\Delta_{f}}{\Delta_{y}}$$

**Table 7:** Ductility calculation.

Specimens	$\Delta_{y}$ (mm)	$\Delta_{u}$ (mm)	$\Delta_{f}$ (mm)	$\mu_{\Delta u}$	$\frac{\mu_{\Delta u}}{{\left(\mu_{\Delta u}\right)}_{CB}}$	$\mu_{\Delta f}$	$\frac{\mu_{\Delta f}}{{\left(\mu_{\Delta f}\right)}_{CB}}$
SB1	18.91	24.6	51	1.30	1.00	2.70	1.00
SB2	17.5	31.95	50	1.83	1.40	2.86	1.06
SB3	19.82	23.91	48	1.21	0.93	2.42	0.90
SB4	18.82	21.32	36	1.13	0.87	1.91	0.71

The reduction in ductility in the strengthened beam is a significant challenge for strengthening systems. Beams SB3 and SB4 exhibit lower ductility compared to reference beam SB1. While SB3 demonstrates relatively better ductility performance than SB4, it still falls short of SB1. The substantial drop in ductility index in SB4 is attributed to brittle failure caused by early premature debonding and concrete cover separation. However, the ductility index ratio in SB2 is slightly better than in SB1 due to the presence of additional reinforcement, which enhances beam ductility without increasing the ultimate load. These observations suggest that the configuration of the proposed system enables the beam to behave similarly to the reference beam.

### 5.3. Failure Mechanism

The selection of bonding between the existing concrete and fresh jacketing concrete surfaces was justified based on experimental findings. Throughout the entire experimental test of the beam, no debonding occurred between these two surfaces from the initial stages until the eventual failure of the beam. Notably, even during and after failure, no debonding took place between these surfaces, proving the effective performance of the strengthened beam.

Different failure modes occurred in the experimental specimens and the FE models. For the strengthened beam SB1, the failure started at the bottom of the beam, with initial cracks developing in the center of the beam, followed by the yielding of the bottom steel bars, and finally failing when the concrete on the top surface reached its limited compressive strength. The absence of concrete jacketing in the SB2 beam prevents the strengthening system from functioning properly, thereby failing to increase the beam’s capacity. Consequently, beams SB1 and SB2 exhibit almost identical performance from the onset of loading to the failure stage. This similarity in behavior demonstrates the ineffectiveness of the proposed strengthening system when concrete jacketing is not applied.

**Figure 14:**
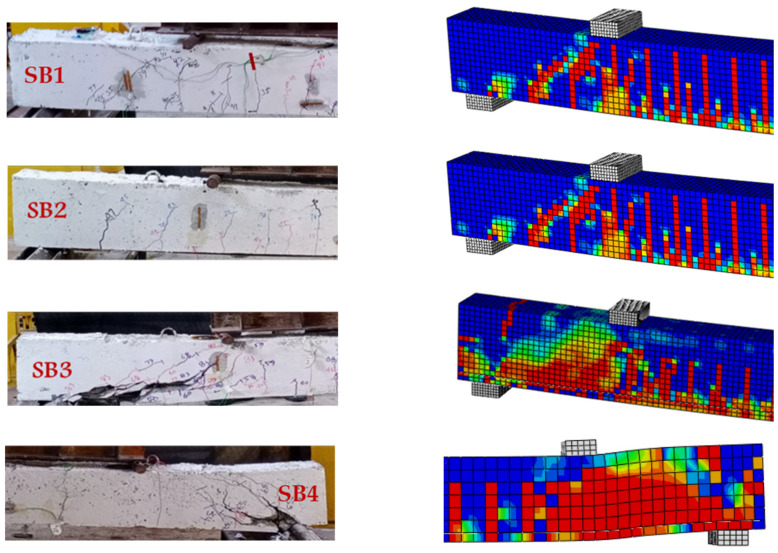
Validation of the failure mechanism of the FE models with the experimental specimens.

Meanwhile, the failure mechanism was entirely different on the SB3. Only tiny initial cracks appeared in the middle part of SB3 compared with SB1, with most of them starting to appear in the span between the point load and the pinned support. These cracks developed and propagated in a diagonal direction at 45 degrees until total failure occurred. This finding indicates that the proposed system can be used to change the failure mechanism for strengthened beams from flexural failure to shear diagonal failure. The SB4 model typically follows the pattern of premature debonding failure at a displacement of around 38 mm. This early debonding deactivated the strengthening system, and a sudden brittle failure occurred at that point. Also, the failure occurred near the roller support. Figure 14 shows the experimental failure side by side with the FE model failure for further validation and comparison. Figure 14 presents detailed images of the failure zone of the strengthened beam during experimental testing. Notably, the strengthening system, including the CFRP bars and MAS, exhibits a negligible impact on SB2. The absence of concrete jacketing hinders the system’s contribution to beam resistance in this case. Also, in SB2, the anticipated sliding of the CFRP bars during loading, particularly when the bottom surface of the beam deflects and exerts pressure on the CFRP bars at the midsection, was observed as expected.

**Figure 15:**
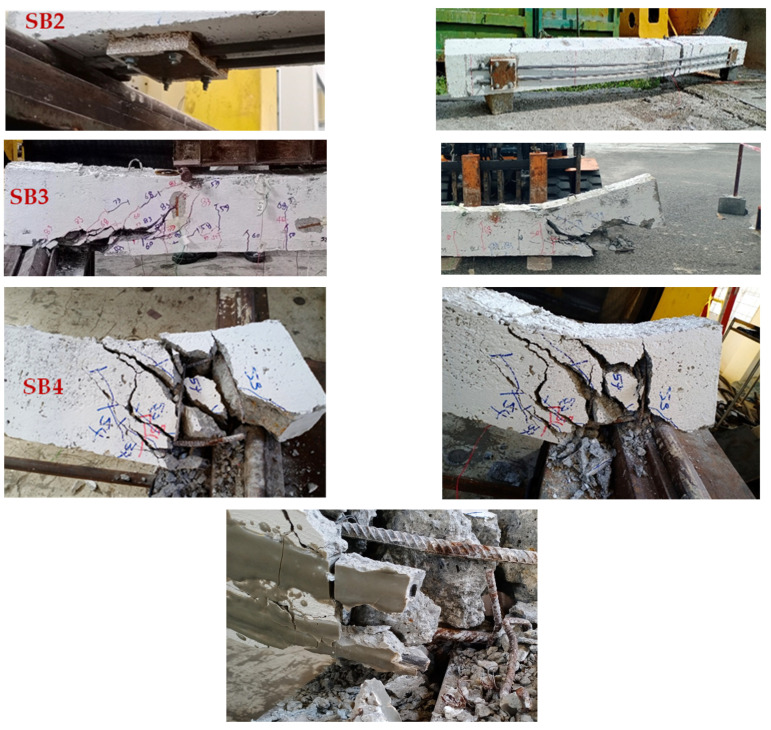
Close-up photos depicting the area of failure in the strengthened beam.

Conclusive evidence of sliding was observed post-beam failure, as depicted in the photograph in Figure 15. In SB3, despite the beam’s destruction due to shear cracking propagation, the MAS with CFRP bars remains intact without crushing failure in the rods. The most significant damage is observed in SB4, where the CFRP debonded from the beam, transferring all stress back to the steel bar, resulting in fracture. The failure photos of SB4 show that the epoxy remained intact around the CFRP rods until the end of the experiment, indicating that bond failure did not occur at the interface between the CFRP and the epoxy. Therefore, it can be inferred that the premature bond failure occurred at the epoxy–concrete interface.

### 5.4. Strain Response

The maximum strain recorded during the experimental test and obtained from FE simulation output results is listed in Table 8. This table presents several findings. The value of the strain gauge in the two steel bars, or even in the two CFRP bars in the experimental work, was slightly different due to the difficulty in symmetrizing the beam configuration or the applied load, but these differences are still within the acceptable limit. The strain in the steel bar in SB3 and SB4 is less than half of its value in SB1 and SB2.

The existence of CFRP bars on SB3 and SB4 reduces the stress transfer into the steel bars, which reduces their deformation and lowers their strain value. The value recorded in the CFRP bars in SB2 was a negligible value close to zero, which indicates that the CFRP bars in SB2 do not involve improving beam capacity due to the absence of concrete jacketing.

Furthermore, the highest strain on CFRP bars was reported in SB3, which means the current system enhances the effective use of CFRP to improve beam resistance. Finally, the difference between the value obtained from the experimental work and FE simulation was generally less than 10%, thus proving the ability of the FE model to predict the beam behavior; except in SB4, it was 42.7% for CFRP bars due to the limited ability of the program to predict the premature debonding failure during the experimental test.

**Table 8:** Maximum strain at ultimate load.

Experimental Work							FE Simulation		Difference	
	At Steel Bars %			At CFRP Bars %			At Steel Bar	At CFRP Bar	At Steel Bar	At CFRP Bar
	Bar 1	Bar 2	Average	Bar 1	Bar 2	Average	%	%	%	%
SB1	0.3941	0.4125	0.4033	-	-	-	0.4398	-	9.1	-
SB2	0.3632	0.3712	0.3672	-	-	-	0.3884	-	5.8	-
SB3	0.1863	0.1782	0.1823	0.3421	0.3678	0.3550	0.1725	0.3536	5.3	3.9
SB4	0.1724	0.8323	0.1778	0.2214	0.1984	0.2099	0.1823	0.2831	2.5	42.7

## 6. Parametric Study

This section investigates the effects of several design parameters on the strengthened beams’ performance. The effect and number of the MASs in the jacketing were studied under a repetitive incremental half-cyclic load. However, the effect of the thickness and grade of the concrete jacketing and the effect of the diameters of the CFRP bars were observed under incremental static loads because the vibration load does not directly affect them so much.

### 6.1. Effect of Number of Employed MASs

**Figure 16:**
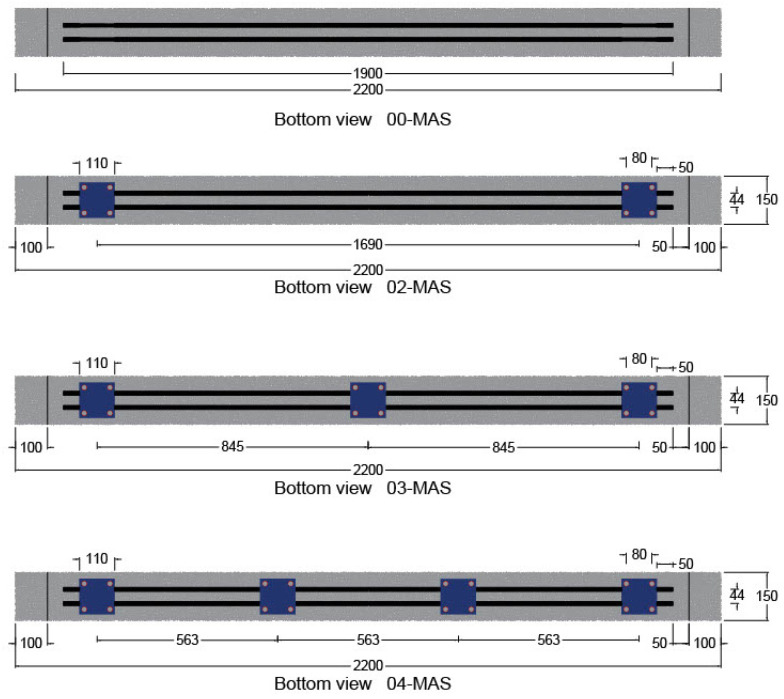
Distribution of MASs at the bottom surface of the beam.

Four models were developed to evaluate the impact of the mechanical anchorage system under half-cyclic loading conditions, as illustrated in Figure 16. The first model (00-MAS) employed no MAS so that its effect on beam performance can be examined. The other three models were equipped with two (02-MAS) units, similar to those used in SB3. The third model (03-MAS) had three MAS units, and the fourth model (04-MAS) had four MAS units so that the influence of increasing their numbers can be assessed.

**Figure 17:**
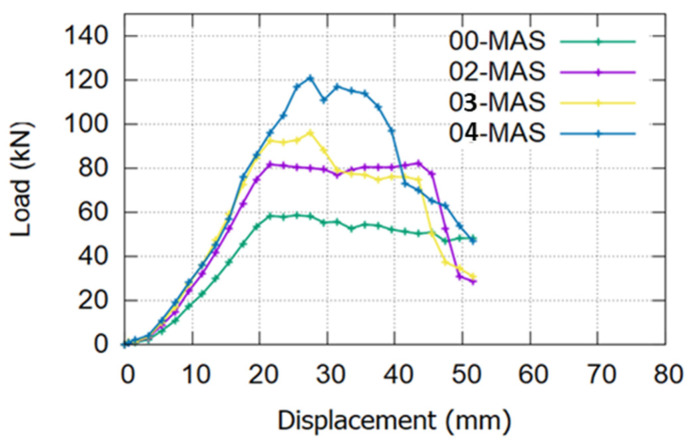
Effect of the number of MASs.

As shown in Figure 17, the FE result proved the MASs’ effectiveness in the proposed system. The increase in the number of MASs increased the strength capacity of the strengthened beam significantly. However, this relationship is not linear; the ultimate load value increased by around 38% when the number of MASs increased from zero to two. Moreover, with three MASs, the ultimate load increased by 19% to reach 97 kN, and with four MASs, it increased by approximately 23% to 121 kN. Increasing the number of MASs enables the CFRP bars to resist more loading, reducing the debonding risk and the fatigue loading effect.

### 6.2. Effect of CFRP Bar Diameter

**Figure 18:**
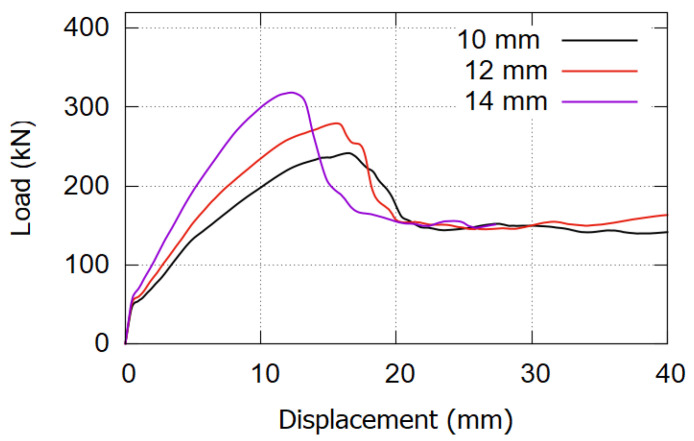
Effect of the CFRP bar diameter.

The effect of the CFRP rod’s diameter on the strengthening system was demonstrated by evaluating the effect of three different CFRP rod diameters. Three almost similar FE models were generated, except that they had different CFRP rod diameters of 10, 12, and 14 mm. All three models were tested under static loads. The load versus displacement curve results are presented in Figure 18.

The CFRP diameter affects the beam’s performance by increasing the amount of reinforcement in the strengthened beam cross-section. As the CFRP cross-section increased from 157, 226, and 307 mm^2^, the ultimate load increased to 243, 271, and 312 kN, respectively. Increasing the area of CFRP bars twice (using 14 mm rods instead of 10 mm) caused the ultimate value to increase by only 28%, from 243 to 312 kN. This result shows that the amount of CFRP material in the section does not highly affect the strengthening system unless there is a chance to effectively use the high strength of the CFRP material. Increasing the amount of CFRP leads to a decrease in the stresses developed on it, which does not help in using the CFRP material effectively. In most conditions, local failure occurs in the steel bar and concrete materials, and the CFRP material does not even reach half its tensile capacity.

### 6.3. Effect of the Concrete Jacketing Parameters

**Figure 19:**
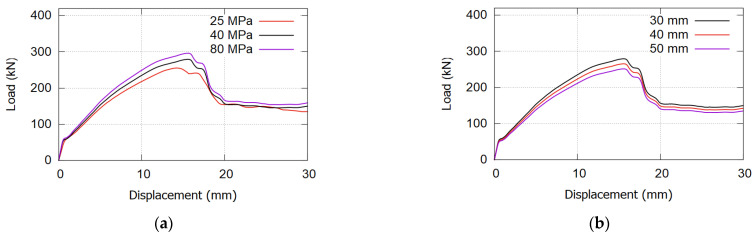
Effect of the concrete jacketing parameters: (**a**) effect of jacketing grade; (**b**) effect of the jacketing thickness.

Figure 19 demonstrates how variations in concrete jacketing parameters impact the overall performance of the strengthened beam. Two parameters were studied: jacketing thickness and concrete grade. Six models were created, each representing different combinations: three concrete grades (25, 40, and 80 MPa) and three thicknesses (30, 40, and 50 mm).

The FE modeling results in Figure 19a reveal the minimal impact of concrete jacketing grade on overall beam performance. Despite varying concrete grades from 25 MPa to 80 MPa, the change in ultimate load was within 10 kN. Notably, the effect was marginal, with 80 MPa slightly increasing and 25 MPa slightly decreasing compared with the proposed 40 MPa. The thin layer of concrete jacketing (30 mm thickness) relative to the beam’s concrete (250 mm thickness) explains the modest improvement in ultimate load with higher-grade jacketing. In addition, higher concrete grades showed no significant influence on the initial crack appearance or yielding of bottom steel reinforcement. Consequently, the use of high-grade concrete jacketing is not recommended due to its limited effect.

Figure 19b reveals an unexpected finding from the FE analysis: increasing jacketing thickness resulted in decreased beam performance. Although the reduction in peak load value ranged from 10% to 15%, it was notable. This outcome is attributed to the thicker concrete jacketing shifting the section’s natural axis downward, impacting beam behavior. Moreover, it adds to the beam’s dead weight and amplifies stresses on the bonding layer between the beam and fresh concrete jacketing.

## 7. Ultimate Load Prediction

Predicting the ultimate load of strengthened beams is crucial in retrofitting. Established techniques such as EB or NSM methods are widely studied by organizations such as the ACI Committee 440 and companies such as Sika. As new retrofitting techniques emerge, researchers often adjust existing empirical equations to suit them. This study adopted the guidelines from ACI-440-2R-17 [5] for ultimate load calculations, as it is the most widely used standard in the field. The flexural capacity of the strengthened beams was determined using limit state principles, ensuring strain and internal force equilibrium across the cross-section. In addition, CFRP bars were assumed to act as additional reinforcement, with the CFRP material demonstrating linear-elastic stress–strain behavior until failure and no relative slip between the CFRP bar and concrete. These assumptions were listed in ACI 440.2R-17 Section 10.2.1 [5] to streamline calculations and facilitate the application of empirical equations. While acknowledging the need for sufficient experimental data to ensure the reliability of predictions, any inaccuracies in these assumptions are not expected to significantly affect the computed flexural strength. The equation used and the calculation steps are summarized as follows:

- Step 1: Determine the design material properties.

Typically, manufacturer-reported material properties do not consider long-term environmental exposure. According to ACI 440.2R-17 C.9.4, CFRP material properties are taken as initial values. These properties should be adjusted based on exposure conditions using the environmental reduction factor in ACI 440.2R-17 Table 9.4 [5]. For carbon fiber and interior exposure, the reduction factor is 0.95. However, in this system, the effect of the reduction factor is assumed to be negligible due to the presence of concrete jacketing, providing full protection from environmental exposure.

The design tensile, strain tensile, and modulus of elasticity for CFRP were calculated according to Equation (11), which is analogous to Equations (9.4a), (9.4b), and (9.4c) in ACI 440.2R-17 [5], respectively.

(11) ffu=CEffu∗εfu=CEεfu∗Ef=ffuεfu

- Step 2: Determine the preliminary calculation.

$\beta_{1}$ (ratio of the depth of the equivalent rectangular stress block to the depth of the neutral axis) is determined according to the equation in ACI 440.2R-17 Section 22.2.2.4.3 [5] of ACI 318-19, as reflected by Equation (12).

(12) β1=1.05−0.05fc′6.9

Also, the $E_{c}$ (modulus of elasticity) of the concrete used for the beam and the concrete jacketing is determined based on the formula in ACI 318-19, as shown in Equation (13)

(13) $$E_{c}=4700\sqrt{f_{c}^{\prime }}$$

- Step 3: Determine the strain on the bottom surface of the beam.

The actual strain on the bottom surface of the beam (*εbi*) was disregarded in the current calculations due to the absence of loading prior to strengthening, coupled with the low self-weight of the beam. Equation (14) is presented below.

(14) $$\varepsilon_{bi}=\frac{M_{DL}\left(d_{f}-C\right)}{I_{cr}E_{c}}$$

- Step 4: Determine the CFRP system bond-dependent coefficient ($\kappa_{m}$).

The dimensionless bond-dependent flexural coefficient ($\kappa_{m}$) for the CFRP bars was set to 0.7 based on manufacturers’ recommendations, as outlined in Equation (15).

(15) $$\kappa_{m}=0.7$$

- Step 5: Assume the distance (C), the depth to the neutral axis.

In the analysis stage, an assumed value was given to the distance (C). A logical assumption helps reduce the number of checks and trial-and-error methods. A reasonable initial estimate for (C) can be taken between 0.15 and 0.20 from the effective depth (d). In this study, it was taken as 0.2 d in Equation (16). This assumed value needs to be modified and adjusted to achieve force equilibrium.

(16) C=0.2 d

- Step 6: Determine the system’s failure mechanism by calculating the effective strain at the CFRP reinforcement bar level.

The strain in CFRP materials dictates the stress developed in the CFRP, as CFRP materials exhibit linear elastic behavior until failure. Failure mechanisms can be identified at points where concrete crushes, CFRP ruptures, or CFRP debonds from the substrate. Equation (10.5.2) from ACI 440.2R-17 [5] can be effectively used to determine the strain in CFRP reinforcement at the ultimate limit condition, as indicated in Equation (17).

If the beam fails due to CFRP debonding from the system, then the ultimate value is obtained by multiplying the design rupture strain of CFRP reinforcement $\left(\varepsilon_{fd}\right)$ by the bond-dependent coefficient ($\kappa_{m}$), which was previously determined in step 4. If the failure mechanism was the rupture of the CFRP bars, then the value of $\left(\varepsilon_{fe}\right)$ was equal to ${(\varepsilon }_{fu}$), determined from CFRP material testing. On the basis of ACI 318-19 [59], the maximum usable strain of unconfined concrete ($\varepsilon_{cu}$) can be taken as 0.003.

(17) $$\varepsilon_{fe}=\varepsilon_{cu}\left(\frac{d_{f}-C}{C}\right)-\varepsilon_{bi}\leq K_{m}\varepsilon_{fd}\;\varepsilon_{fe}=\varepsilon_{cu}\left(\frac{d_{f}-C}{C}\right)-\varepsilon_{bi}\;\varepsilon_{fe}=K_{m}\varepsilon_{fd}$$

The failure mechanism for all the strengthened beams with the proposed system was concrete crushing in compression. Hence, Equation (18) can be utilized to calculate the strain in the compressed concrete, which exceeded its ultimate limit value of 0.003.

(18) $$\varepsilon_{c}=\left(\varepsilon_{fd}+\varepsilon_{bi}\right)\left(\frac{d_{f}-C}{C}\right)$$

- Step 7: Determine the strain in the primary steel reinforcement.

The strain in the steel reinforcement can be determined using the triangular similarity of the strain graph and the known value obtained in the previous step, as depicted in Equation (19).

(19) $$\varepsilon_{s}=\left(\varepsilon_{fe}+\varepsilon_{bi}\right)\left(\frac{d-C}{d_{f}-C}\right)$$

- Step 8: Determine the stress levels for the main steel reinforcement and the additional CFRP reinforcement.

The main steel reinforcement exhibits elastic behavior up to the yielding point, while the CFRP maintains elastic behavior until failure. Equations (20) and (21) were utilized to compute their stress levels, utilizing their previously determined respective strain values, applying Hooke’s law as follows:

(20) $$f_{s}=E_{s}\varepsilon_{s}\leq f_{y}$$

(21) $$f_{fe}=E_{f}\varepsilon_{fe}$$

- Step 9: Determine approximate concrete stress block factors.

On the compression side, concrete stress block factors can be determined using the parabolic stress–strain relationship, as shown in Equations (22) and (23). Alternatively, these factors can be determined using ACI 318.

(22) $$\alpha_{1}=\frac{3\overset{´}{\varepsilon_{c}}\varepsilon_{c}-{\varepsilon_{c}}^{2}}{3\beta_{1}{\overset{´}{\varepsilon_{c}}}^{2}}$$

(23) $$\beta_{1}=\frac{4\overset{´}{\varepsilon_{c}}-\varepsilon_{c}}{6\overset{´}{\varepsilon_{c}}-2\varepsilon_{c}}$$

where ($\overset{´}{\varepsilon_{c}}$) is the compressive strain of unconfined concrete corresponding to the specified compressive strength of the concrete ($f_{c}^{\prime })$ and may be taken as 0.002 or calculated by using Equation (24).

(24) $$\overset{´}{\varepsilon_{c}}=\frac{1.7f_{c}^{\prime }}{E_{c}}$$

- Step 10: Determine the internal force acting on the strengthened cross-section and check force equilibrium.

Equation (25) illustrates three forces in the strengthened section at the ultimate load state: two tension forces and one compression force. Force equilibrium requires the sum of all forces on the section to equal zero. Force equilibrium is confirmed by validating the initially estimated C value.

(25) $$F_{c}=F_{s}+F_{f}\;\alpha_{1}f_{c}^{\prime }\beta_{1}bC=A_{s}f_{s}+A_{f}f_{fe}\;C=\frac{A_{s}f_{s}+A_{f}f_{fe}}{\alpha_{1}f_{c}^{\prime }\beta_{1}b}$$

- Step 11: Adjust the value of C until force equilibrium is achieved.

The value of C in step 4 is reassumed. Then, all parameters on the sections are recalculated and force equilibrium is verified iteratively until the value of C achieves the required force equilibrium

- Step 12: Determine the components of flexural strength.

The flexural strength of the strengthened cross-section arises from two components. Equations (26) and (27) demonstrate the contribution to bending for both steel reinforcement and CFRP bar reinforcement, respectively.

(26) $$M_{ns}=A_{s}f_{s}\left(d-\frac{\beta_{1}C}{2}\right)$$

(27) $$M_{nf}=A_{f}f_{fe}\left(d_{f}-\frac{\beta_{1}C}{2}\right)$$

- Step 13: Determine the design flexural strength of the strengthened section.

Equation (28) represents the ultimate design flexural strength of the entire section of the strengthened beams. This equation applies additional reduction factors ${(\varphi }_{f}$) to account for the contribution of CFRP materials to the flexural strength.

(28) $$M_{n}=M_{ns}+\varphi_{f}M_{nf}$$

**Figure 20:**
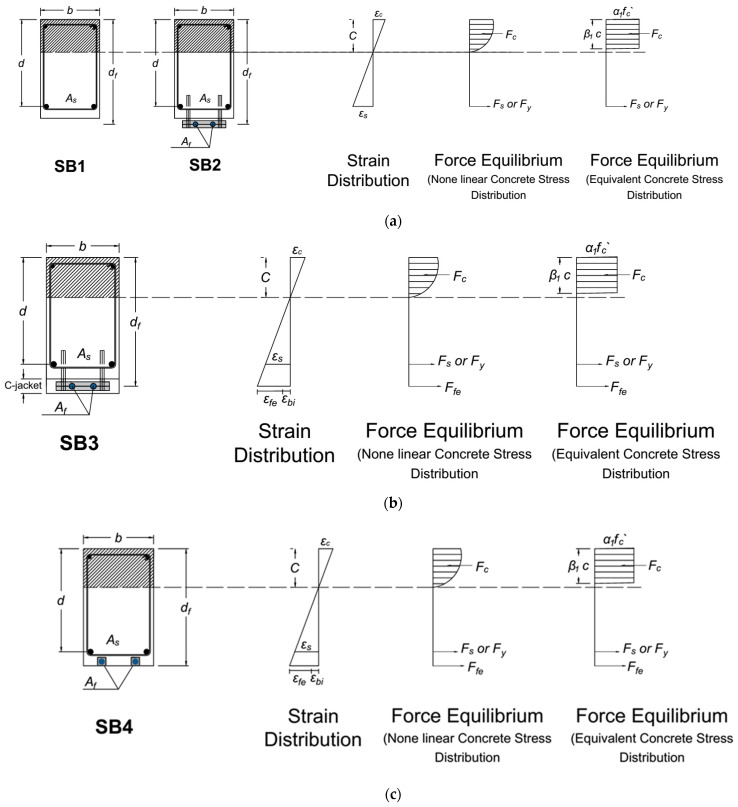
Internal strain, stress, and force equilibrium for the beam’s section: (**a**) reference beam SB1 and strengthened beam SB2; (**b**) strengthened beam SB3; (**c**) strengthened beam SB4.

SB1 (reference beam) was initially analyzed as a rectangular, single-reinforced beam. SB2 underwent the same analysis, as the lack of concrete cover precluded any enhancement in beam capacity (Figure 20a). In SB3, section dimensions increased due to concrete jacketing, with tensile force originating from both steel reinforcement and CFRP bars (Figure 20b). Lastly, SB4’s analysis followed the conventional NSM approach (Figure 20c).

Table 9 outlines the output results for the beams, presenting the design flexural strength of the section ($\emptyset M_{n}$ [kN.m]) and its equivalent ultimate load ($P_{u}$[kN]) in the second and third columns, respectively. The fourth column demonstrates the effect of the strengthening system on enhancing the beam capacity. The system’s effect was calculated based on the value of the predicted ultimate load on the strengthened beams compared to its value in the reference beam.

The equations that are utilized to determine the flexural strength of the strengthened system assume full bonding and disregard loading and other boundary conditions. Their analysis solely considers the 2D section, resulting in SB1 and SB2 having the same value of 17.88 kN.m (as the additional reinforcement was not factored into the calculation due to the absence of concrete jacketing). Due to the additional CFRP rod reinforcement in the cross-sectional plan, SB3 demonstrates a value of 53.88 kN.m. The value for SB4 was 47.89 kN.m due to the implementation of the NSM system.

**Table 9:** Ultimate load prediction.

Items	Calculation under Static		Effect of Strengthening System %
	$\emptyset M_{n}$ (kN.m)	$P_{u}$ (kN)	
SB1	17.88	71.51	-
SB2	17.88	71.51	-
SB3	53.83	215.30	201
SB4	47.89	191.54	168

## 8. Conclusions

The key findings of this study are summarized as follows:

- The proposed system effectively prevented premature debonding, with no instances in the strengthened beam from the initiation of loading until failure. This resulted in a substantial improvement in the beam’s capacity under vibration load, increasing from 44 kN in SB1 to 83 kN in SB3. In contrast, the beam strengthened using the conventional NSM technique experienced a capacity increase to 61 kN only primarily due to early debonding issues.
- The presence of an intermediary medium is crucial for effectively transferring stress and load from the existing beam to the attachment-strengthening system. Hence, employing the proposed system without concrete jacketing hinders the CFRP bars from effectively enhancing beam performance.
- The proposed strengthening system, like conventional EB and NSM methods, aims to enhance beam flexural capacity. This results in a change in the failure mechanism in the beam from flexural to shear failure due to the presence of sufficient reinforcement to resist shear under high loading conditions.
- Increasing the CFRP bar diameter does not uniformly enhance the beam capacity. This observation stemmed from the failure mechanism, where collapse ensued due to the development of shear failure combined with steel yielding followed by compression concrete crushing, irrespective of the CFRP bar diameter. In addition, in all models, CFRP material stresses remained significantly distant from local damage.
- Increasing the thickness of the concrete jacketing adversely affects the beam’s ultimate load primarily due to the accompanying increase in dead load and stresses in the epoxy layer. In addition, while the concrete’s grade significantly impacts the initial crack value, its effect on the final ultimate load is negligible due to the relatively small amount of concrete in the jacketing compared with that in the beam itself.
- ACI 440.2R-17 offers a conservative estimate of ultimate load capacity for beams under static load but lacks guidance for predicting behavior under varied loading conditions, such as incremental cyclic loading, as utilized in this work. The results underscore the significant impact of vibration on reducing the capacity of the strengthened beam.
- Design guidelines often rely on cross-section analysis to determine capacity, overlooking the influence of bonding or anchorage in the entire beam system. This approach neglects the structural anchorage and bonding conditions, making it unreliable to rely solely on analytical calculations.

### Recommendations for Future Research

- The combination of two strengthening systems to enhance beam flexural and shear performance should be explored. This approach would address the transformation from flexural failure to shear failure, which occurs when the strengthening system fails to prevent the formation of critical shear cracks.
- Additional design parameters related to the beam’s properties, such as variations in beam width, height, and reinforcement ratio, should be investigated.
- The MAS design, particularly in combination with FRP strips or sheets, should be investigated to improve practical applications.

## Data Availability

The original contributions presented in the study are included in the article, further inquiries can be directed to the corresponding author.

## List of Notations

$A_{f}$	area of FRP external reinforcement (mm^2^)	$M_{cr}$	cracking moment (N-mm)
$A_{s}$	area of non-prestressed steel reinforcement (mm^2^)	$M_{DL}$	moment due to dead load (N-mm)
a	depth of equivalent concrete compression block	$M_{n}$	nominal flexural strength (N-mm)
b	short-side dimension of compression member of	$M_{nf}$	contribution of FRP reinforcement to nominal
	prismatic cross-section.		flexural strength (N-mm)
c	distance from extreme compression fiber to the	$n_{f}$	modular ratio of elasticity between FRP and
	neutral axis (mm)		concrete $E_{f}/E_{c}$
$C_{E}$	environmental reduction factor	$n_{s}$	modular ratio of elasticity between FRP and
d	distance from extreme compression fiber to centroid		concrete $E_{s}/E_{c}$
	of tension reinforcement (mm)	$\alpha_{1}$	ratio of the depth of equivalent rectangular stress
$d_{f}$	effective depth of FRP flexural reinforcement (mm)		block to depth of the neutral axis
$E_{c}$	modulus of elasticity of concrete (MPa)	$\beta_{1}$	ratio of the depth of equivalent rectangular
$E_{f}$	tensile modulus of elasticity of FRP (MPa)		block to depth of the natural axis
$E_{s}$	modulus of elasticity of steel (MPa)	$\in_{bi}$	strain in the concrete substrate at the time of FRP
$F_{c}$	compression force acting on concrete		installation (tension is positive)
$F_{f}$	tension force acting on FRP material	$\in_{c}$	strain in concrete
$F_{f}$	tension force acting on steel reinforcement	$\in_{c}^{\prime }$	maximum strain of unconfined concrete
$f_{c}^{\prime }$	specified compressive strength of concrete (MPa)		corresponding to fc
$f_{f}$	stress in FRP reinforcement	$\in_{cu}$	ultimate axial strain of unconfined concrete
$f_{fe}$	effective stress in the FRP; stress level attained at		corresponding to 0.85$f_{co}^{\prime }\;$or maximum usable
	section failure (MPa)		strain of unconfined concrete (mm/mm), which
$f_{fu}$	design ultimate tensile strength of FRP (MPa)		can occur at ${f_{c}=0.85f}_{c}^{\prime }$ or $\in_{c}=0.003$, depending
$f_{fu}^{*}$	ultimate tensile strength of the FRP material as		on stress–strain curve
	reported by the manufacturer (MPa)	$\in_{f}$	strain in FRP reinforcement
$f_{r}$	modulus of rupture of concrete (MPa)	$\in_{fd}$	debonding strain of FRP reinforcement
$f_{s}$	stress in non-prestressed steel reinforcement (MPa)	$\in_{fe}$	effective strain in FRP reinforcement attained at
$f_{y}$	specified yield stress in non-prestressed steel		failure
	reinforcement (MPa)	$\in_{fu}$	design rupture strain of FRP reinforcement
h	overall thickness of height of the member (mm)	$\in_{fu}^{*}$	ultimate rupture strain of FRP reinforcement
$h_{j}$	thickness of concrete jacketing	$\in_{s}$	strain in non-prestressed steel reinforcement
$h_{T}$	overall height of strengthened beam (${h+h}_{j}$)	$k_{m}$	bond-dependent coefficient of the FRP system
$I_{cr}$	moment of inertia of cracked section transformed to	$\psi_{f}$	FRP strength reduction factor = 0.85 for flexure
	concrete (mm^4^)		(calibrated based on design material properties)
k	ratio of the depth of neutral axis to reinforcement		
	depth measured from extreme compression fiber		
